# Role of Contrast Enhancement and Corrected Attenuation Values of Renal Tumors in Predicting Renal Cell Carcinoma (RCC) Subtypes: Protocol for a Triphasic Multi-Slice Computed Tomography (CT) Procedure

**DOI:** 10.12659/PJR.901957

**Published:** 2017-07-16

**Authors:** Ersen Ertekin, Akın Soner Amasyalı, Bulent Erol, Saim Acikgozoglu, Faruk Kucukdurmaz, Alaaddin Nayman, Haluk Erol

**Affiliations:** 1 Department of Radiology, Adnan Menderes University, Faculty of Medicine, Aydın, Turkey; 2 Department of Urology, Adnan Menderes University, Faculty of Medicine, Aydın, Turkey; 3 Department of Urology, Medeniyet University, School of Medicine, Istanbul, Turkey; 4 Department of Radiology, Necmettin Erbakan University, School of Medicine, Konya, Turkey; 5 Department of Urology, Sutcu Imam University, School of Medicine, Kahramanmaras, Turkey; 6 Department of Radiology, Selcuk University, School of Medicine, Konya, Turkey

**Keywords:** Carcinoma, Renal Cell, Contrast Media, Multidetector Computed Tomography

## Abstract

**Background:**

To distinguish RCC subtypes based on contrast enhancement features of CT images.

**Material/Methods:**

In total, 59 lesions from 57 patients were included. All patients underwent multi-slice CT imaging with a triphasic protocol, which included non-contrast, corticomedullary, nephrographic and urographic phases. Contrast enhancement features of renal masses were evaluated in terms of CT attenuation values (AV) and differences in contrast density; the aorta or renal parenchyma were evaluated based on corrected or relative values.

**Results:**

Clear cell RCC (ccRCC) showed more intense contrast enhancement than other RCC subtypes. When differentiating ccRCC from other RCC subtypes, a cut-off AV of 86–89 HU, aorta-based corrected AV of 89–95 HU and renal parenchyma-based corrected AV of 87–95 HU showed a diagnostic accuracy of 81–86%, 86–88% and 74–78%, respectively, in the corticomedullary phase. Furthermore, a cutoff of 2.42–2.72 for the relative contrast enhancement ratio, a cutoff of 2.59–2.74 for the aorta-based corrected relative contrast enhancement ratio and a cutoff of 2.63–2.76 for the renal parenchyma-based attenuation ratio showed a diagnostic accuracy of 83–88%, 88–90% and 81%, respectively.

**Conclusions:**

The most reliable parameters for differentiating ccRCC from other RCC subtypes are aorta-based corrected AV and aorta-based corrected relative contrast enhancement values in the corticomedullary phase.

## Background

Renal cell carcinoma (RCC) is the third most common urogenital cancer and it accounts for 2–3% of all cancers [1]. With a routine use of CT scans for assessing abdominal symptoms, the incidental RCC prevalence has increased. Up to 85% of all RCC cases are clear-cell RCCs (ccRCCs), which is the most aggressive form of RCC in comparison to the papillary (pRCC) and chromophobe (chRCC) subtypes [2]. RCC displays an unpredictable clinical behavior, and it is important to determine its TNM stage, histologic subtype and Fuhrman grade in each case.

Patients with ccRCC have a poorer prognosis than those with other RCC subtypes, which is why a preoperative discrimination between these subtypes is clinically important and useful for generating a personalized treatment plan. Furthermore, active surveillance, biopsy or neoadjuvant therapy may be considered in some cases. For these reasons, the awareness of RCC subtypes is becoming increasingly important. In the modern era, improved imaging techniques help differentiate between renal tumors and their different histologic subtypes. In general, it is accepted that after administration of a contrast agent, tumor enhancement of >15 Hounsfield units (HU) is suggestive of a malignancy [3]. However, this is insufficient for predicting histologic subtypes of RCC and differentiating them from benign tumors such as oncocytoma. The differentiation between RCC subtypes by contrast-enhanced CT has been previously investigated [4]. Kim et al. found that a cut-off value of 84 HU in the corticomedullary phase differentiated ccRCC from other RCC subtypes with a sensitivity and specificity of 74% and 100%, respectively [5]. Ruppert-Kohlmayr et al. reported that aorta-based corrected AV was not affected by intrinsic factors such as cardiac output and therefore gave a cut-off value of 100 HU, with a sensitivity and specificity for discriminating ccRCC in the corticomedullary phase of 95.7% and 98.3%, respectively [6].

It is believed that the nephrographic phase is superior to the corticomedullary phase in distinguishing an intraparenchymal renal mass on enhanced CT [7]. However, the corticomedullary phase provides more information on angiogenesis and vascularization of the renal mass, and it was shown that ccRCC and oncocytoma are easier to characterize in the corticomedullary phase [8]. In our institution, in suspected renal masses, the corticomedullary, nephrographic and urographic phases are performed followed by an unenhanced CT. In this prospective study, we aimed to differentiate the subtypes of RCC with triphasic CT imaging based on contrast enhancement features and AV.

## Material and Methods

This prospective study was approved by the institutional ethics committee and enrolled a total of 57 patients (35 male, 22 female) with 59 renal masses. There were 2 ipsilateral renal masses that existed simultaneously in 2 female patients. Before surgery, all patients underwent triphasic (corticomedullary, nephrographic and urographic) CT scans after unenhanced CT. A Siemens, 64-sequence Somatom Sensation device (Erlangen, Germany) was used for CT scans. Intravenous contrast agent (300/100 mg/ml=30 gr) was administered via the antecubital route by an automated power injector at a rate of 3–4 ml/s. Times after contrast injection of 25 seconds, 1 minute and 5 minutes were accepted as the corticomedullary, nephrographic and urographic phases, respectively. One-millimeter-thick sections were obtained from all images to facilitate the investigation of 3D reconstructions.

Age and gender of the patient, lesion dimensions, existence of calcifications, contrast enhancement and tumoral spread patterns were noted. Radiologic staging was done according to the 2009 TNM classification system. After the procedure, the distribution of the histopathologic subtypes was as follows: 34 – clear cell, 15 – chromophobe, 7 – papillary, 2 – sarcomatoid and 1 – collecting tubular type RCC. The appropriate grade of each tumor was reported using the Fuhrman’s system.

### Imaging analysis

First, all renal masses were evaluated according to their contrast enhancement patterns and were classified as homogeneous or heterogeneous. Subsequently, AVs of the tumors were calculated for each phase. Density measurement was performed by calculating the mean density value of different circular areas (1–2 cm^2^) in the solid tumor. Non-calcified areas and preferentially most hyperenhancing components were chosen for density measurements.

The degree of contrast enhancement may be affected by intrinsic factors such as cardiac output and BMI and therefore we calculated aorta-based corrected AV and renal parenchyma-based corrected or relative values. We used a coefficient factor, which is defined as the ratio of the mean aorta AV of all patients to the patient’s own aorta AV. Multiplying the coefficient factor by the tumor AV gave the aorta-based corrected AV. The aorta AV was measured from the renal artery branching level of the aorta, and this was measured for each phase. The mean aorta AV of all patients was 250 HU, 120 HU and 90 HU for corticomedullary, nephrographic and urographic phases, respectively. Moreover, we calculated relative contrast enhancement values by dividing the corrected AV by tumor AV on unenhanced CT images. The same calculations were also performed to generate renal parenchyma-based corrected or relative values. These values were all noted before surgery and subsequently compared with postoperative histopathologic features. The calculation method of aorta corrected attenuation values and relative enhancement values was similar to that used by Ruppert-Kohlmayr et al. (6).

### Statistical analysis

All data were fed into SPSS 15 software, and descriptive statistics were presented as means ±SD and percentile units. Rare histologic subtypes such as sarcomatoid and collecting tubular RCC type were not included in the analysis. The chi-squared test was used to compare categorical parameters. The Kruskal-Wallis test was used to compare RCC subtypes. A Bonferroni-corrected Mann-Whitney U test was done for pairwise comparisons between three parameters. Contrast enhancement cut-off values were calculated by a ROC curve analysis and crosstabs analysis. P-values below 0.05 were considered to be statistically significant.

## Results

**Figure 1:**
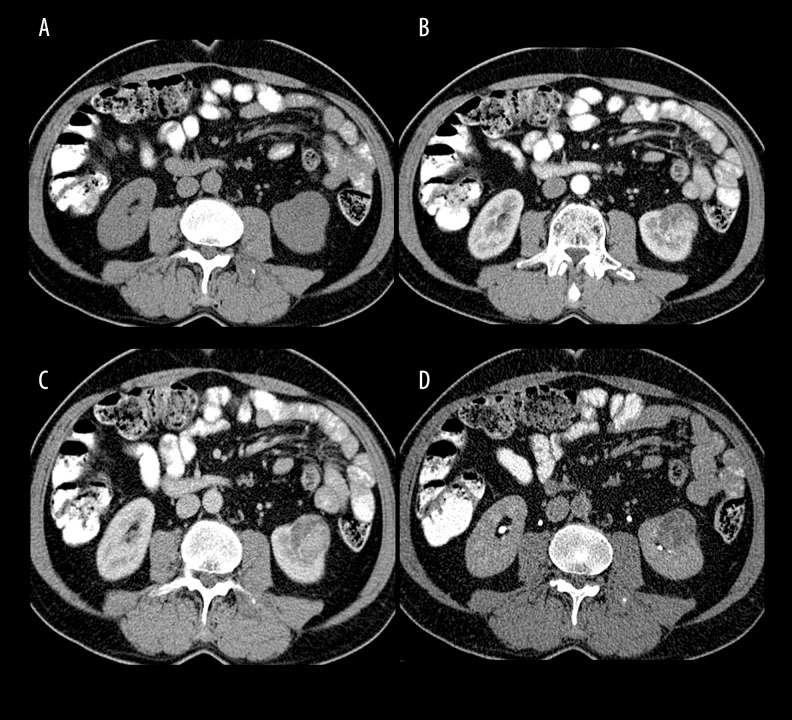
A 51-year-old male patient with clear cell renal cell carcinoma (3.5 cm in diameter). (**A**) A non-contrast image shows well-circumscribed mass causing contour lobulation. (**B**) Corticomedullary phase, cortical mass shows heterogeneous contrast enhancement. Enhancement areas similar to the cortex density are seen in the mass. (**C**) Nephrographic phase, intense heterogeneous enhancement continues in the mass. (**D**) Urographic phase, the relationship with the collecting system of the mass is revealed more clearly.

**Figure 2:**
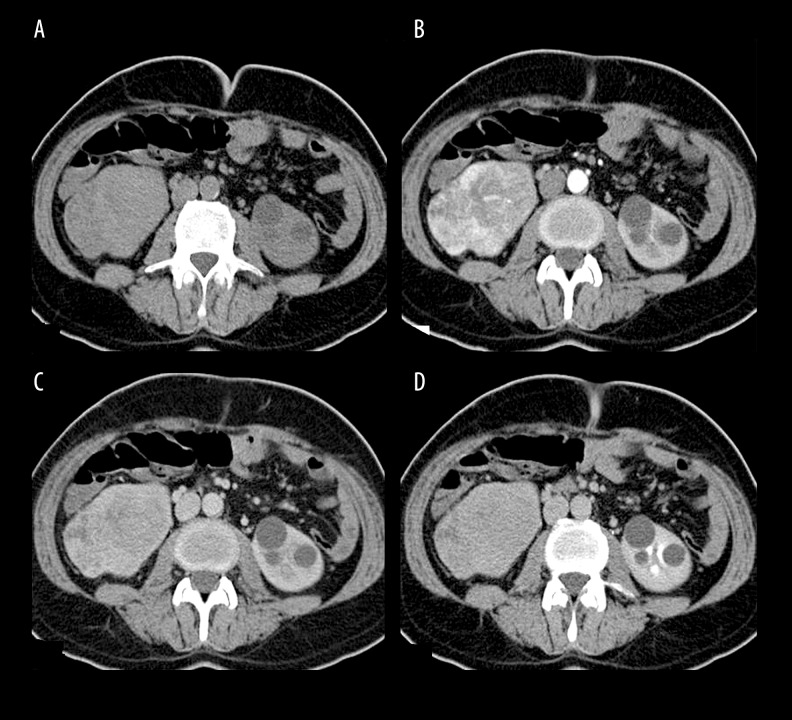
A 44-year-old male patient with chromophobe renal cell carcinoma (9 cm in diameter). (**A**) A smooth, lobulated, isodense mass lesion is observed in the right kidney on non-contrast image. (**B**) Corticomedullary phase, the mass shows heterogeneous enhancement. Perinephric stranding is seen around the lesion (perinephric invasion). (**C**) Heterogeneous enhancement of the lesion and perinephric change was preserved in the nephrographic phase. (**D**) Wash-out of the contrast in the mass lesion is seen in the urographic phase.

**Figure 3:**
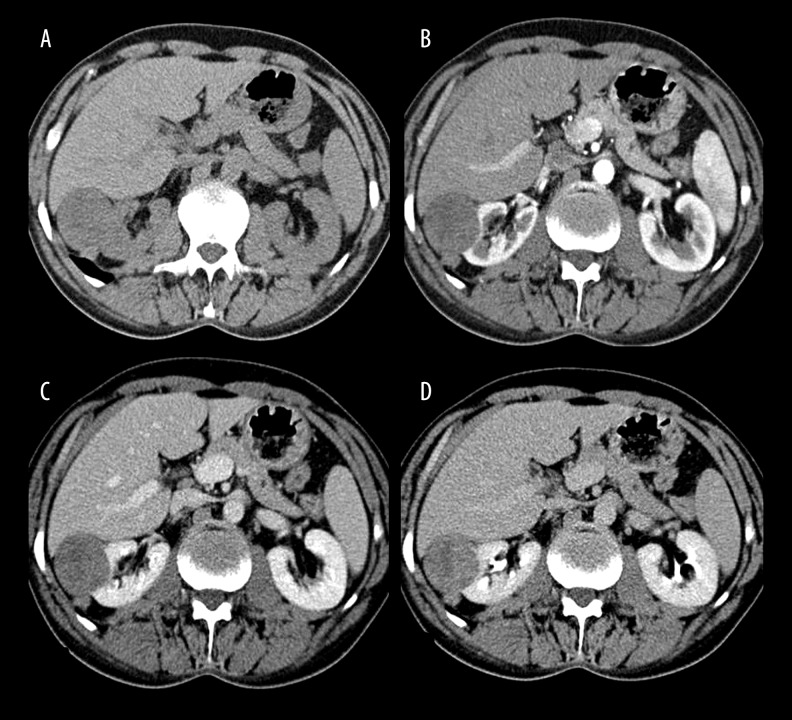
A 59-year-old male patient with papillary renal cell carcinoma (4.5 cm in diameter). (**A**) On non-contrast image, a well-circumscribed right renal cortical mass compressing the liver and a perinephric nodule is observed in the posterior aspect of the mass. (**B**) Corticomedullary phase, in small amounts nearly homogeneous enhancement of the lesion is seen. There is also homogeneous enhancement in the perinephric nodule. (**C**) Nephrographic phase, a slight increase in the enhancement of the mass and perinephric nodule is seen. (**D**) Urographic phase shows clearly the absence of collecting system invasion by the mass lesion.

Fifty-nine renal tumors were investigated in our study. The male-to-female ratio was 1.6, and the mean age was 55.4±11.3 (33–79). The mean tumor diameter was 7.2±4.3 (2–20 cm), and the histologic subtypes were 58% – ccRCC, 25% – chRCC, 12% – pRCC, 3% – sarcomatoid and 2% – collecting tubular type RCC. There were no statistically significant differences between the 3 most prevalent subtypes (ccRCC, chRCC and pRCC) in terms of age, gender or tumor diameter (p>0.05). A heterogeneous contrast enhancement pattern was detected in 94%, 80% and 57% of the ccRCC, chRCC and pRCC cases, respectively. However, the differences were not significant (p>0.05) (Figures 1–3).

**Figure 4:**
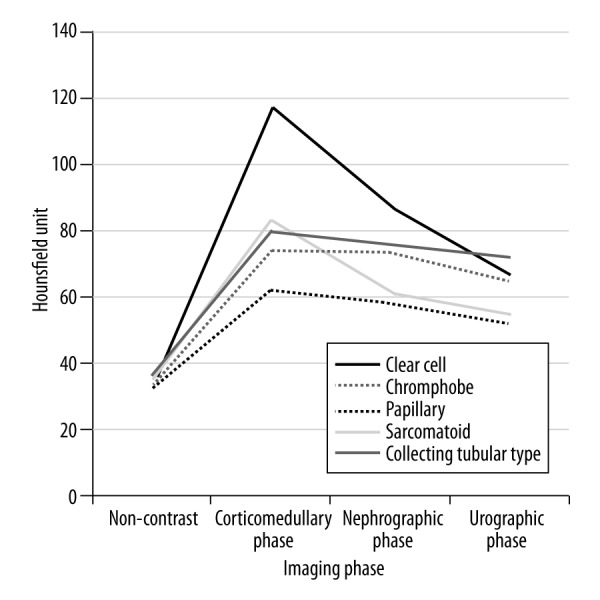
Mean attenuation values of renal tumors on multiphasic computed tomography images.

All tumor AVs are given in Table 1. There was no difference between the 3 subtypes in the unenhanced CT images (p>0.05). However, ccRCC AVs were higher than the respective values of other RCC subtypes in all 3 phases. This difference was significant in both the corticomedullary phase (ccRCC *vs.* chRCC, p<0.017 and ccRCC *vs.* pRCC, p<0.017) and the nephrographic phase (ccRCC *vs.* pRCC, p<0.017 and ccRCC *vs.* chRCC, p<0.018) but was not significant in the urographic phase (p>0.05). There was no difference between chRCC and pRCC in terms of tumor AVs (p>0.05) (Figure 4).

Similarly, the aorta-based corrected AV was also higher in ccRCC compared to chRCC and pRCC in the corticomedullary phase (p<0.01). This significant difference between ccRCC and pRCC (p=0.014) persisted in the nephrographic phase. However, the difference between the three subtypes in the aorta-based corrected AVs was not statistically significant in the urographic phase (p>0.05). The same significant difference was found between the three subtypes in terms of renal parenchyma-based corrected AVs in all 3 phases (Table 2). In addition, the relative values were calculated based on aorta and renal parenchyma in the three phases, and the aorta-based corrected relative contrast enhancement values are given in Table 3.

In the ROC curve analysis, the cut-off values with the highest sensitivity and specificity were determined in the aorta-based corrected AV and aorta-based corrected relative contrast enhancement values in the corticomedullary phase for differentiation between ccRCC and other RCC subtypes. A cut-off aorta-based corrected AV of 91 HU showed an accuracy rate of 88%, positive predictive value of 91%, negative predictive value of 85%, sensitivity of 88% and specificity of 88%, respectively. Moreover, a cutoff of 2.59 of the aorta-based corrected relative contrast enhancement value showed a sensitivity, specificity, positive predictive value, negative predictive value and accuracy rate of 97%, 80%, 87%, 95% and 90%, respectively.

## Discussion

Currently, choosing treatment for renal tumors, such as active surveillance, more aggressive treatment (e.g., minimally invasive interventional methods and partial nephrectomy) or neoadjuvant targeted therapy, depends on the patient’s prognosis and the tumor’s histologic subtype [9]. RCC subtypes have different prognoses, and patients with ccRCC have the poorest prognosis. The five-year overall survival rates are 55–60%, 80–90% and 90% for ccRCC, pRCC and chRCC, respectively [10,11]. Thus, differentiating RCC subtypes preoperatively is important for generating a personalized treatment plan.

Several retrospective studies have been conducted that examined certain enhancement features of renal tumors that could potentially discriminate between RCC subtypes [4–6,12,13]. In general, these studies have shown that significantly higher attenuation values were indicative of ccRCC. Zhang et al. concluded that an increased and earlier enhancement of ccRCC and oncocytoma was due to their hypervascular formations [14]. Furthermore, Jinzaki et al. found a correlation between high microvascular density and increased tumor AV (>100 HU) in ccRCC patients with a tumor diameter of <3.5 cm [15]. In contrast, our prospectively designed, triphasic multi-slice CT protocol study examined aorta-based corrected AVs and relative values; it demonstrated that the aforementioned parameters were more valuable than was tumor AV alone for predicting ccRCC preoperatively.

Sheir et al. reported that a heterogeneous contrast enhancement pattern was more commonly seen in ccRCC [4]. However, this result was not statistically significant. Similarly, we observed a heterogeneous contrast enhancement pattern more frequently, particularly in patients with ccRCC <4 cm. Furthermore, Pierorazio et al. investigated renal tumors (<4 cm) and found the highest peak HU value in the corticomedullary phase for ccRCC (117 HU), in the nephrographic phase for oncocytoma (125 HU) and in the nephrographic phase for pRCC (56 HU) [16]. This finding indicates that a more aggressive approach may be preferred in renal tumors with a higher HU value in the corticomedullary phase and a heterogeneous contrast enhancement pattern that is smaller than 4 cm.

Contrast enhancement pattern may be influenced by several factors such as BMI, cardiac output or renal function [17]. Therefore, other indicators are also required for subtype differentiation. Kohlmayr et al. noted that aorta-based corrected AVs could eliminate the influence of intrinsic factors [6]. They found that a cutoff of 100 HU of the aorta-based corrected AV could differentiate ccRCC in the corticomedullary phase with sensitivity of 95.7% and specificity of 98.3%. In contrast, we reported a cut-off value of 91 HU for the same parameter with sensitivity of 88% and specificity of 88%. Moreover, the same study found sensitivity of 94.5% and specificity of 75% when 2.0 was accepted as the aorta-based relative contrast enhancement cut-off value, whereas we reported a cut-off value of 2.59 for the same parameter with sensitivity of 97% and specificity of 80%. We assume that these different results may be explained by the amount of contrast agent that was used in each study (120 ml *vs.* 100 ml).

Small renal masses are more likely to be benign in comparison to larger ones. It was reported that 22% of all surgically resected masses sized 2–3 cm were benign [18]. Chaudhry et al. used only unenhanced CT attenuation measurements and concluded that they could not reliably differentiate minimal fat renal angiomyolipoma from RCC in small renal masses [19]. However, recently published data have shown that ccRCC could be discriminated from pRCC, chRCC and lipid-poor angiomyolipoma with the ratio of tumor to kidney AV in the nephrographic phase, whereas oncocytoma overlapped with both ccRCC and chRCC. However, the corticomedullary phase was not provided in their study [20]. In contrast, in the present study, we used all three phases for evaluation but did not assess the discrimination of RCC from benign renal masses. In addition, another retrospective study investigated the differentiation between RCC subtypes by the tumor-to-aorta enhancement ratios in the corticomedullary phase. Unfortunately, the authors did not detect any significant difference between ccRCC and oncocytoma [21].

More recently, a study by Mazzei et al. revealed that CT perfusion parameters (surface permeability, mean transit time, blood volume and blood flow of the tumor) may help differentiate RCCs from oncocytomas. Furthermore, that study claimed that CT perfusion parameters could provide additional information to multiphasic CT for subtype differentiation [22]. However, further studies with larger numbers of patients are still needed to validate the CT perfusion technique. We did not use CT perfusion in our study, but the enhancement patterns were similar to the aforementioned data. Finally, the present study has some limitations. Our study population was limited, and benign renal lesions such as oncocytoma or angiomyolipoma were not evaluated. Moreover, measurements were done by the same radiologist without any observer. However, preoperatively recorded data made the study prospective in character, and both the radiologist and pathologists were blinded.

## Conclusions

It is well established that the ccRCC subtype shows more intense contrast enhancement in comparison to other malignant forms of RCC. We demonstrated that the most reliable parameters for the differentiation of the clear cell subtype from other RCC subtypes are the aorta-based corrected AVs and the aorta-based corrected relative contrast enhancement values in the corticomedullary phase. In the future, this may impact treatment decisions for small renal masses, if the high accuracy rates in this study are supported by studies with larger cohorts.

## Figures and Tables

**Table 1:** Mean contrast enhancement values and statistical data.

	ccRCC	chRCC	pRCC	sRCC	cttRCC
**CMP** (HU)	85±31 (44–157)	41±23 (20–108)	30±12 (16–51)	54 (53–55)	44
**NP** (HU)	55±18 (26–97)	40±19 (22–94)	26±9 (14–40)	27 (26–27)	40
**UP** (HU)	34±13 (16–61)	31±13 (18–64)	20±9 (9–33)	21 (17–24)	36
	**CMP**		**NP**		**UP**
**ccRCC-chRCC**	**P=0.000**		p=0.021		P=0.152
**ccRCC-pRCC**	**P=0.000**		**P=0.000**		P=0.067
**chRCC-pRCC**	P=0.298		P=0.162		P=0.358

ccRCC – clear cell renal cell carcinoma; chRCC – chromophobe renal cell carcinoma; pRCC – papillary renal cell carcinoma; sRCC – sarcomatoid renal cell carcinoma; cttRCC – collecting tubular type renal cell carcinoma; CMP – corticomedullary phase; NP – nephrographic phase; UP – urographic phase.

**Table 2:** Mean aorta and renal parenchyma-based corrected attenuation values and statistical data.

	Aorta-based corrected AV			Renal parenchyma-based corrected AV		
	CMP	NP	UP	CMP	NP	UP
**ccRCC** (HU)	121±33 (75–213)	88±21 (46–135)	66±11 (45–93)	122±47 (74–267)	90±25 (62–160)	72±17 (40–112)
**chRCC** (HU)	74±25 (44–84)	78±24 (42–136)	63±14 (42–124)	86±32 (43–154)	75±18 (48–108)	63±12 (44–84)
**pRCC** (HU)	63±15 (42–90)	61±16 (40–85)	53±14 (33–80)	61±16 (48–96)	54±12 (42–75)	51±12 (36–69)
**sRCC** (HU)	87 (82–93)	71 (66–75)	55 (54–56)	89 (86–92)	62 (59–65)	54 (46–62)
**cttRCC** (HU)	79	68	61	87	75	75
	**Aorta-based corrected AV**			**Renal parenchyma-based corrected AV**		
	**CMP**	**NP**	**UP**	**CMP**	**NP**	**UP**
**ccRCC-chRCC**	**P=0.000**	P=0.162	P=0.276	**P=0.002**	P=0.077	P=0.135
**ccRCC-pRCC**	**P=0.000**	**P=0.004**	P=0.085	**P=0.000**	**P=0.000**	P=0.074
**chRCC-pRCC**	P=0.407	P=0.106	P=0.368	P=0.078	P=0.021	P=0.055

ccRCC – clear cell renal cell carcinoma; chRCC – chromophobe renal cell carcinoma; pRCC – papillary renal cell carcinoma; sRCC – sarcomatoid renal cell carcinoma; cttRCC – collecting tubular type renal cell carcinoma; CMP – corticomedullary phase; NP – nephrographic phase; UP – urographic phase; AV – attenuation values.

**Table 3:** Mean aorta-based relative enhancement values (aREV) and statistical data.

	ccRCC	chRCC	pRCC	sRCC	cttRCC
aREV CMP	3.6±0.15 (2.1–6.0)	2.2±0.20 (1.4–4.1)	2.0±0.15 (1.3–2.6)	2.5±0.16 (2.3–2.7)	2.2
aREV NP	2.7±0.11 (1.3–4.1)	2.4±0.22 (1.1–4.8)	1.9±0.14 (1.5–2.4)	2.0±0.12 (1.9–2.1)	1.9
aREV UP	2.0±0.07 (1.3–3.0)	1.9±0.11 (1.1–2.5)	1.6±0.11 (1.3–2.1)	1.6±0.03 (1.5–1.6)	1.7
	**CMP**		**NP**		**UP**
**ccRCC-chRCC**	**P=0.000**		P=0.077		P=0.145
**ccRCC-pRCC**	**P=0.000**		**P=0.003**		P=0.096
**chRCC-pRCC**	P=0.783		P=0.078		P=0.489

ccRCC – clear cell renal cell carcinoma; chRCC – chromophobe renal cell carcinoma; pRCC – papillary renal cell carcinoma; sRCC – sarcomatoid renal cell carcinoma; cttRCC – collecting tubular type renal cell carcinoma; CMP – corticomedullary phase; NP – nephrographic phase; UP – urographic phase.

## References

[1] Capitanio U, Cloutier V, Zini L (2009). A critical assessment of the prognostic value of clear cell, papillary and chromophobe histological subtypes in renal cell carcinoma: a population-based study. BJU Int.

[2] Keegan KA, Schupp CW, Chamie K (2012). Histopathology of surgically treated renal cell carcinoma: survival differences by subtype and stage. J Urol.

[3] Israel GM, Bosniak MA (2008). Pitfalls in renal mass evaluation and how to avoid them. Radiographics.

[4] Sheir KZ, El-Azab M, Mosbah A (2005). Differentiation of renal cell carcinoma subtypes by multislice computerized tomography. J Urol.

[5] Kim JK, Kim TK, Ahn HJ (2002). Differentiation of subtypes of renal cell carcinoma on helical CT scans. Am J Roentgenol.

[6] Ruppert-Kohlmayr AJ, Uggowitzer M, Meissnitzer T, Ruppert G (2004). Differentiation of renal clear cell carcinoma and renal papillary carcinoma using quantitative CT enhancement parameters. Am J Roentgenol.

[7] Johnson PT, Horton KM, Fishman EK (2010). How not to miss or mischaracterize a renal cell carcinoma: protocols, pearls, and pitfalls. Am J Roentgenol.

[8] Gakis G, Kramer U, Schilling D (2011). Small renal oncocytomas: differentiation with multiphase CT. Eur J Radiol.

[9] Ljungberg B, Cowan CC, Hanbury DC (2010). EAU guidelines on renal cell carcinoma: The 2010 update. Eur Urol.

[10] Lam JS, Klatte T, Kim HL (2008). Prognostic factors and selection for clinical studies of patients with kidney cancer. Crit Rev Oncol Hematol.

[11] Karakiewicz PI, Suardi N, Capitanio U (2009). A preoperative prognostic model for patients treated with nephrectomy for renal cell carcinoma. Eur Urol.

[12] Herts BR, Coll DM, Novick AC (2002). Enhancement characteristics of papillary renal neoplasms revealed on triphasic helical CT of the kidneys. Am J Roentgenol.

[13] Young JR, Margolis D, Sauk S (2013). Clear cell renal cell carcinoma: discrimination from other renal cell carcinoma subtypes and oncocytoma at multiphasic multidetector CT. Radiology.

[14] Zhang J, Lefkowitz RA, Ishill NM (2007). Solid renal cortical tumors: Differentiation with CT. Radiology.

[15] Jinzaki M, Tanimoto A, Mukai M (2000). Double-phase helical CT of small renal parenchymal neoplasms: Correlation with pathologic findings and tumor angiogenesis. J Comput Assist Tomogr.

[16] Pierorazio PM, Hyams ES, Tsai S (2013). Multiphasic enhancement patterns of small renal masses (≤4 cm) on preoperative computed tomography: Utility for distinguishing subtypes of renal cell carcinoma, angiomyolipoma, and oncocytoma. Urology.

[17] Platt JF, Reige KA, Ellis JH (1999). Aortic enhancement during abdominal CT angiography: Correlation with test injections, flow rates, and patient demographics. Am J Roentgenol.

[18] Frank I, Blute ML, Cheville JC (2003). Solid renal tumors: An analysis of pathological features relat- ed to tumor size. J Urol.

[19] Chaudhry HS, Davenport MS, Nieman CM (2012). Histogram analysis of small solid renal masses: Differentiating minimal fat angiomyolipoma from renal cell carcinoma. Am J Roentgenol.

[20] Ishigami K, Pakalniskis MG, Leite LV (2015). Characterization of renal cell carcinoma, oncocytoma, and lipid-poor angiomyolipoma by unenhanced, nephrographic, and delayed phase contrast-enhanced computed tomography. Clin Imaging.

[21] Zokalj I, Marotti M, Kolarić B (2014). Pretreatment differentiation of renal cell carcinoma subtypes by CT: the influence of different tumor enhancement measurement approaches. Int Urol Nephrol.

[22] Mazzei FG, Mazzei MA, Cioffi Squitieri N (2014). CT perfusion in the characterisation of renal lesions: An added value to multiphasic CT. Biomed Res Int.

